# *Toxoplasma gondii* Infection in Dustmen in Northeastern China: A Case-Control Seroprevalence Study

**DOI:** 10.1155/2018/3207675

**Published:** 2018-12-19

**Authors:** Ruo-Lan Jiang, Quan Zhao, Jing Jiang, Xue-Long Chen, Xiao-Xuan Zhang, Xiang Wu

**Affiliations:** ^1^Department of Parasitology, Xiangya School of Medicine, Central South University, Changsha, Hunan Province 410013, China; ^2^College of Animal Science and Technology, Changchun Sci-Tech University, Shuangyang, Jilin Province 130600, China; ^3^College of Animal Science and Veterinary Medicine, Heilongjiang Bayi Agricultural University, Daqing, Heilongjiang Province 163319, China

## Abstract

**Background:**

Toxoplasmosis is caused by an intracellular parasite* Toxoplasma gondii*, which can infect many hosts including humans.

**Methods:**

In order to estimate whether dustmen are more susceptible to* T. gondii*, a case-control study was conducted containing 332 dustmen from Jilin and Heilongjiang in Northeastern China, as well as 332 general populations from the same regions as control subjects. Serum samples were tested IgG and IgM antibodies to* T. gondii* using the enzyme-linked immunosorbent assay (ELISA).

**Results:**

The overall anti-*T. gondii* IgG was 15.06% (50/332) in dustmen compared with 9.64% (32/332) in the controls (*P* = 0.0337). Also, 5 (1.51%) dustmen had anti-*T. gondii* IgM antibodies compared with 2 (0.60%) control individuals (*P* = 0.2543). A significant association was only found between dustmen and level of* T. gondii* IgG in comparison with the control subjects. Seroprevalence of* T. gondii* IgG antibodies in male dustmen was significant higher than male control subjects (*P* = 0.0399). Dustmen from Jilin had the significant higher* T. gondii* IgG rate (*P* = 0.0143), in comparison with the control subjects from Jilin. Moreover, dustmen raising cat at home had the significant higher* T. gondii* IgG rate (*P* = 0.0097), in comparison with the control subjects. Risk factor analysis suggested that raising cat at home and not having habits of washing hand before eating were mainly related to the* T. gondii* infection in dustmen.

**Conclusions:**

This is the first record of seroprevalence of* T. gondii* infection in dustmen in Jilin and Heilongjiang provinces in Northeastern China. These findings also suggest that the government departments should pay close attention to the toxoplasmosis in dustmen in Northeastern China.

## 1. Introduction

Toxoplasmosis is a zoonotic disease caused by an intracellular protozoan parasite* Toxoplasma gondii*, which has a global distribution and wide-ranges of hosts [1, 2]. Felines are the only definitive hosts for the parasite and virtually all warm-blooded animals are intermediate hosts such as humans [3, 4]. Foodborne and waterborne transmission as well as transplacental infection are the major routes for transmission of* T. gondii* [5, 6]. Although 1/3 of the world populations are seropositive for* T. gondii*, most of them are asymptomatic [3]. However, it can cause various severe diseases, and even death in immunocompromised individuals [7].

In view of such severe situations, it is essential to investigate the status of* T. gondii* infection in workers in different professions. There are some investigations focusing on detection of* T. gondii* in people worked at different professions recently. For example, Alvarado-Esquivel et al. demonstrated that 23 (12.0%) of 192 truck drivers were anti-*T. gondii* IgG antibodies positive in Mexico [8]; moreover, a total of 278 out of 464 migrant workers have been detected as Sahimin et al. detected as* T. gondii*-positive in Malaysia [9]. The similar studies have also been conducted in China, such as involving in livestock and poultry breeding and processing workers [10]. Dustman is an occupation prone to acquire several infections and toxoplasmosis in one of them [11]. It is important that information regarding* T. gondii* infection in dustmen is still scarce [11], especially in China. Therefore, a case-control study was conducted containing 332 dustmen from Jilin and Heilongjiang in Northeastern China, as well as 332 general populations from the same regions as control subjects, to determine the seroprevalence of* T. gondii* in dustmen and to estimate whether dustmen are more susceptible to be* T. gondii*-infected compared with general populations in Jilin and Heilongjiang in Northeastern China.

## 2. Materials and Methods

### 2.1. Study Design and Population

The study was approved by the Changchun Sci-Tech University. Participants were made aware of the aim of the study. A total of 664 blood samples (including 332 dustmen and 332 control subjects) were collected from Jilin Province (41°N–46°N, 122°E–131°E) and Heilongjiang Province (43°26′N–53°33′N, 121°11′E–135°05′E) in Northeastern China, January 2017 and March 2018. The individuals' occupations and names were not recorded to ensure confidentiality. The purposes and procedures of the study were explained to all participants, and written informed consent was obtained from them all. The sera were collected with agreements from the volunteers. Control sera were collected from volunteers. Information regarding gender, age, whether raising cat at home, whether having habits of washing hands before meals, or geographic region was recorded.

### 2.2. Sample Collection and Serological Tests

About 5 mL of venous blood was collected aseptically from each participant in Eppendorf tubes and kept at room temperature for 2 h. Then serum was separated from the whole blood by centrifugation at 3,000 rpm for 10 min, which was labeled and frozen at -20°C until use. Testing for* T. gondii *serology (IgG and IgM) was performed using commercial enzyme immunoassay kits (Demeditec Diagnostics GmbH, Germany) [4]. Positive, negative, and blank controls were included in every plate. Optical densities were measured by photometer at a wavelength of 450 nm. Values higher than the cut-off (10 IU/mL) were considered positive.

### 2.3. Date Analysis

The data of questionnaire covered information such as gender, age, whether raising cat at home, whether having habits of washing hands before meals, or geographic region. The information of questionnaire and experimental results was entered on to an excel spreadsheet and transferred to SPSS v. 19.0 software package (SPSS Inc., USA) [4]. Univariate analysis was used to analyze the association between variables and* T. gondii *infection. Probability (P) value < 0.05 was considered as statistically significant in the analysis.

## 3. Results and Discussion

In the present study, a total of 332 dustmen and 332 control individuals were examined (Tables 1 and 2). Of the dustmen, 50 (15.06%) and 5(1.51%) were detected as anti-*T. gondii* IgG and IgM antibodies positive, respectively, based on the ELISA methods (Tables 1 and 2). Of them, only two samples were detected as both anti-*T. gondii* IgG and IgM antibodies positive. The overall prevalence of anti-*T. gondii *IgG and IgM antibodies in the control individuals was 9.64% (32/332) and 0.60% (2/332), respectively (Tables 1 and 2). The* T. gondii* IgG seropositivity rate was 15.70% (17/147) and 16.08% (23/185) in dustmen in Jilin and Heilongjiang, respectively (Table 1). There are 8.67% (13/150) and 10.44% (19/182) control individuals were examined as* T. gondii* IgG seropositivity in Jilin and Heilongjiang, respectively (Table 1). The dustmen (15.06%) had the significant higher* T. gondii* IgG seroprevalence than control subjects (9.64%,* P* = 0.0337) (Table 1); however, no significant difference of* T. gondii* IgM seroprevalence was found between dustmen group and control group (*P* = 0.2543) (Table 2). In dustmen groups, having cat at home (*P* = 0.0269) and not having habits of washing hand before eating (*P* = 0.0117) were identified to be associated with* T. gondii* infection in dustmen (Table 1).

*T. gondii* is one of the most important foodborne zoonotic pathogens, which can cause various severe diseases and even death in immunocompromised individuals [7]. Our case-control study firstly estimated the seroprevalence of* T. gondii* infection in dustmen. A total of 50 out of 332 dustmen were examined as* T. gondii* IgG-positive. The overall seroprevalence of anti-*T. gondii* IgG was 15.06%, which is significant higher than that in control individuals (9.64%, 32/332,* P* = 0.0337). However, although the seroprevalence of anti-*T. gondii* IgM in dustmen was higher than control groups, the difference was not statistically significant (*P* = 0.2543). These findings suggest that dustmen are more susceptible to be* T. gondii*-infected than general individuals, and the government departments should pay close attention to the toxoplasmosis in dustmen.

Previous studies have also demonstrated that age was highly related to the* T. gondii* seropositivity [12–15]. Perhaps, elder dustmen had more opportunity to interact with the infective oocysts than younger dustmen. In the present study, seroprevalence of* T. gondii* in dustmen was increase with age (Table 1); however, the difference was not statistically significant (*P* = 0.6599). Moreover, previous studies demonstrated that females were more susceptible to the males, which differ from the present results, revealing that no significant difference in the* T. gondii* IgG prevalence between males (15.43%) and females (14.71%) was found (Table 1), and males had a significant higher* T. gondii* IgM prevalence than females (*P* = 0.0210) (Table 2). Also, there was no significant difference statistically among different area of residence (Tables 1 and 2). However, in geographic region groups, dustmen from Jilin had the significant higher* T. gondii* IgG seroprevalence (*P* = 0.0143) (Table 1) than control subjects from Jilin; the findings suggest that dustmen from Jilin must be must be paid attention to detection of* T. gondii *infection.

Cat is the only definitive host for* T. gondii*, so they play a crucial role in transmitting* T. gondii *[3, 16, 17]. Cats discharge feces containing* T. gondii *oocysts into the environment and then become the potential resources of human infection [18]. Therefore, dustmen raising cat at home had a significant higher* T. gondii* IgG seroprevalence than not raising cat at home groups. Moreover, both* T. gondii* IgG and IgM seroprevalence in dustmen who have habits of washing hands before eating were significant lower than those who have not habits of washing hands before eating. The findings suggest the dustmen and other people must cultivate good health habits and wash hands before eating.

## 4. Conclusion

In summary, this is the first report of seroprevalence of* T. gondii *infection in dustmen in Jilin and Heilongjiang Province in Northeastern China. Dustmen were seen to be more susceptible than control individuals. Seroprevalence of* T. gondii* infection in dustmen was mainly related to raising cat at home and not washing hands before eating. The findings will provide key and baseline data for prevention and control of toxoplasmosis in dustmen.

## Figures and Tables

**Table 1:** Seroprevalence of* T. gondii* IgG infection in dustmen and control subjects in China.

Characteristic	Dustmen				Control subjects				Dustmen vs Controls
	No. tested	No. positive	% (95% CI)	*P *value	No. tested	No. positive	% (95% CI)	*P *value	*P* value
Age Group (years)									
≤ 40	24	4	16.67 (0.59-32.74)	0.6599	112	12	10.71 (4.90-16.53)	0.8646	0.4115
41 – 50	129	22	17.05 (10.48-23.63)		82	8	8.89 (2.90-14.88)		0.1390
51 – 60	179	24	18.32 (11.61-25.03)		138	12	8.70 (3.94-13.46)		0.1898
Gender									
Male	162	25	15.43 (9.81-21.05)	0.8533	171	14	8.19 (4.04-12.34)	0.3557	0.0399
Female	170	25	14.71 (9.33-20.08)		161	18	11.18 (6.26-16.10)		0.3403
Geographic region									
Jilin	147	27	15.70 (10.21-21.19)	0.1331	150	13	8.67 (4.11-13.22)	0.5859	0.0143
Heilongjiang	185	23	16.08 (9.99-22.18)		182	19	10.44 (5.95-14.92)		0.5488
Cat at home									
No	154	16	10.39 (5.52-15.26)	0.0269	137	13	9.49 (4.52-14.46)	0.9383	0.7980
Yes	178	34	19.77 (13.76-25.78)		195	19	9.74 (5.54-13.94)		0.0097
Habits of washing hand before meals									
No	158	28	16.28 (10.71-21.85)	0.0117	161	22	13.66 (8.30-19.03)	0.0159	0.3190
Yes	174	22	14.47 (8.81-20.13)		171	10	5.85 (2.30-9.40)		0.0686
Total	332	50	15.06 (11.19-18.93)		332	32	9.64 (6.45-12.83)		0.0337

**Table 2:** Seroprevalence of *T. gondii *IgM infection in dustmen and control subjects in China.

Characteristic	Dustmen				Control subjects				Dustmen vs Controls
	No. tested	No. positive	% (95% CI)	*P* value	No. tested	No. positive	% (95% CI)	*P* value	*P* value
Age Group (years)									
≤ 40	24	0	0 (-)	0.8173	112	1	0.89 (0-2.66)	0.7103	0.6422
41 – 50	129	2	1.55 (0-3.71)		82	0	0 (-)		0.2573
51 – 60	179	3	1.68 (0-3.57)		138	1	0.72 (0-2.16)		0.4518
Gender									
Male	162	5	3.09 (0.39-5.78)	0.0210	171	2	1.17 (0-2.80)	0.1560	0.2230
Female	170	0	0 (-)		161	0	0 (-)		-
Geographic region									
Jilin	147	2	1.36 (0-3.26)	0.8462	150	1	0.67 (0-1.98)	0.8907	0.5499
Heilongjiang	185	3	1.62 (0-3.46)		182	1	0.55 (0-1.63)		0.3226
Cat at home									
No	154	2	1.30 (0-3.11)	0.7730	137	0	0 (-)	0.2345	0.1836
Yes	178	3	1.69 (0-3.59)		195	2	1.03 (0-2.45)		0.5800
Habits of washing hand before meals									
No	158	5	3.16 (0.41-5.92)	0.0181	161	2	1.24 (0-2.97)	0.1438	0.2413
Yes	174	0	0 (-)		171	0	0 (-)		-
Total	332	5	1.51 (0.19-2.82)		332	2	0.60 (0-1.44)		0.2543

## Data Availability

Data on which this article was written could be available as innominate data upon request and approval from Changchun Sci-Tech University.
